# Loss of Function of *AFG3L2* Leading to Developmental and Epileptic Encephalopathy

**DOI:** 10.1002/cns.71013

**Published:** 2026-07-07

**Authors:** Zou Pan, Li Yang, Haiyan Tang, Chen Chen, Qian Yang, Tenghui Wu, Fang He, Zhanwei Zhang, Fangyun Liu, Jing Peng

**Affiliations:** ^1^ Department of Pediatrics Xiangya Hospital of Central South University Changsha China; ^2^ Clinical Research Center for Children's Neurodevelopmental Disabilities of Hunan Province, Xiangya Hospital of Central South University Changsha China; ^3^ NHC Key Laboratory of Human Stem Cell and Reproduction Engineering, Xiangya School of Basic Medical Sciences Central South University Changsha China

**Keywords:** *AFG3L2*, developmental and epileptic encephalopathy, genomic and transcriptomic sequencing, m‐AAA protease, mitochondrial dysfunction

## Abstract

**Aim:**

To delineate the clinical features of *AFG3L2*‐related developmental and epileptic encephalopathy (DEE) and explore its pathogenic mechanisms.

**Methods:**

Whole‐genome and blood transcriptome sequencing were performed in undiagnosed DEE patients. Patient‐derived skin fibroblasts were established for the analysis of RNA and protein expression as well as for mitochondrial functional assays, including OPA1 processing, mtDNA copy number, membrane potential, ATP production, mitochondrial morphology analysis, and mitochondrial stress testing. Additionally, published *AFG3L2*‐related epilepsy cases were systematically reviewed.

**Results:**

We identified four novel *AFG3L2* variants in four DEE patients from two unrelated families, including splice‐site/intronic variants in one family and exon‐deletion/intronic variants in the other, fitting a recessive model of disease. In these patients, plus six additional previously reported DEE patients, symptoms included severe developmental delay, intractable seizures, microcephaly, generalized spasticity, and progressive cerebral atrophy. Transcriptome and fibroblast functional analyses revealed aberrant splicing, reduced *AFG3L2* expression, defective OPA1 processing, decreased mtDNA content, impaired membrane potential and ATP production, fragmented mitochondrial networks, and diminished respiratory capacity, supporting a loss‐of‐function mechanism. Compared with spastic ataxia 5—usually involving null‐missense or missense‐missense genotypes—DEE predominantly features null‐null combinations.

**Significance:**

We implicate *AFG3L2* as a novel causative gene for DEE, likely through mitochondrial proteostasis failure and bioenergetic compromise, expanding the phenotypic and genotypic spectrum of *AFG3L2*‐related disorders.

## Graphical abstract

Biallelic loss‐of‐function AFG3L2 variants define a severe DEE phenotype with microcephaly, spasticity, and progressive cerebral atrophy. Multi‐omics and patient fibroblasts reveal aberrant splicing, reduced AFG3L2, defective OPA1 processing, mtDNA depletion, fragmented mitochondria, and impaired respiration, implicating m‐AAA protease failure in early neurodevelopment.

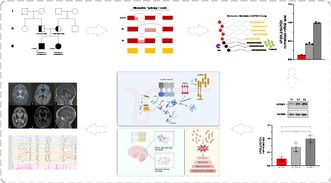

## 1 Introduction

The *AFG3L2* gene encodes AFG3‐like protein 2, a core subunit of the matrix ATPases associated with diverse cellular activities (m‐AAA) protease complex, which forms homo‐oligomeric or hetero‐oligomeric complexes with paraplegia, encoded by *SPG7* [1, 2]. The m‐AAA protease is critical for maintaining mitochondrial homeostasis through protein quality control, processing, and maturation, all of which are essential for neuronal survival [3, 4]. Loss of AFG3L2 function may compromise m‐AAA protease activity and disrupt protein quality control within the inner mitochondrial membrane, thereby predisposing neurons to mitochondrial dysfunction and neurodegeneration [5, 6].

Pathogenic variants in *AFG3L2* underlie a spectrum of neurodegenerative disorders with distinct genotype–phenotype correlations. Heterozygous variants affecting the ATPase domain are associated with optic atrophy 12 (OPA12; MIM #618977), whereas heterozygous variants in the proteolytic domain cause spinocerebellar ataxia type 28 (SCA28; MIM #610246). Biallelic variants affecting the proteolytic domain result in a more severe recessive phenotype, spastic ataxia 5 (SPAX5; MIM #610245). More recently, a few studies have reported that patients with bi‐allelic pathogenic variants in *AFG3L2* can present with myoclonic seizures or epileptic spasms [7, 8, 9, 10]. However, the relationship between *AFG3L2* and epilepsy remains unclear.

In this study, we identified four novel compound heterozygous variants in the intronic regions of *AFG3L2* through whole‐genome sequencing (WGS) in four patients from two unrelated families, all of whom presented with a novel, non‐OMIM phenotype. Subsequently, we performed whole‐blood RNA sequencing and functional experiments on patient‐derived skin fibroblasts to validate the pathogenicity of these variants and to explore their potential mechanism.

## 2 Materials and Methods

Detailed materials and methods are provided in the (Data S1) and describe the following: clinical data collection; WGS and RNA sequencing; bioinformatic analysis of WGS and RNA‐seq data; isolation and culture of fibroblast cell lines; assessment of *AFG3L2* mRNA and protein levels by quantitative real‐time polymerase chain reaction and Western blotting; analysis of mitochondrial morphology; quantification of mitochondrial DNA copy number; assessment of ATP production and mitochondrial membrane potential (MMP); and measurement of mitochondrial oxygen consumption.

## 3 Results

### 3.1 Clinical Information

**FIGURE 1:**
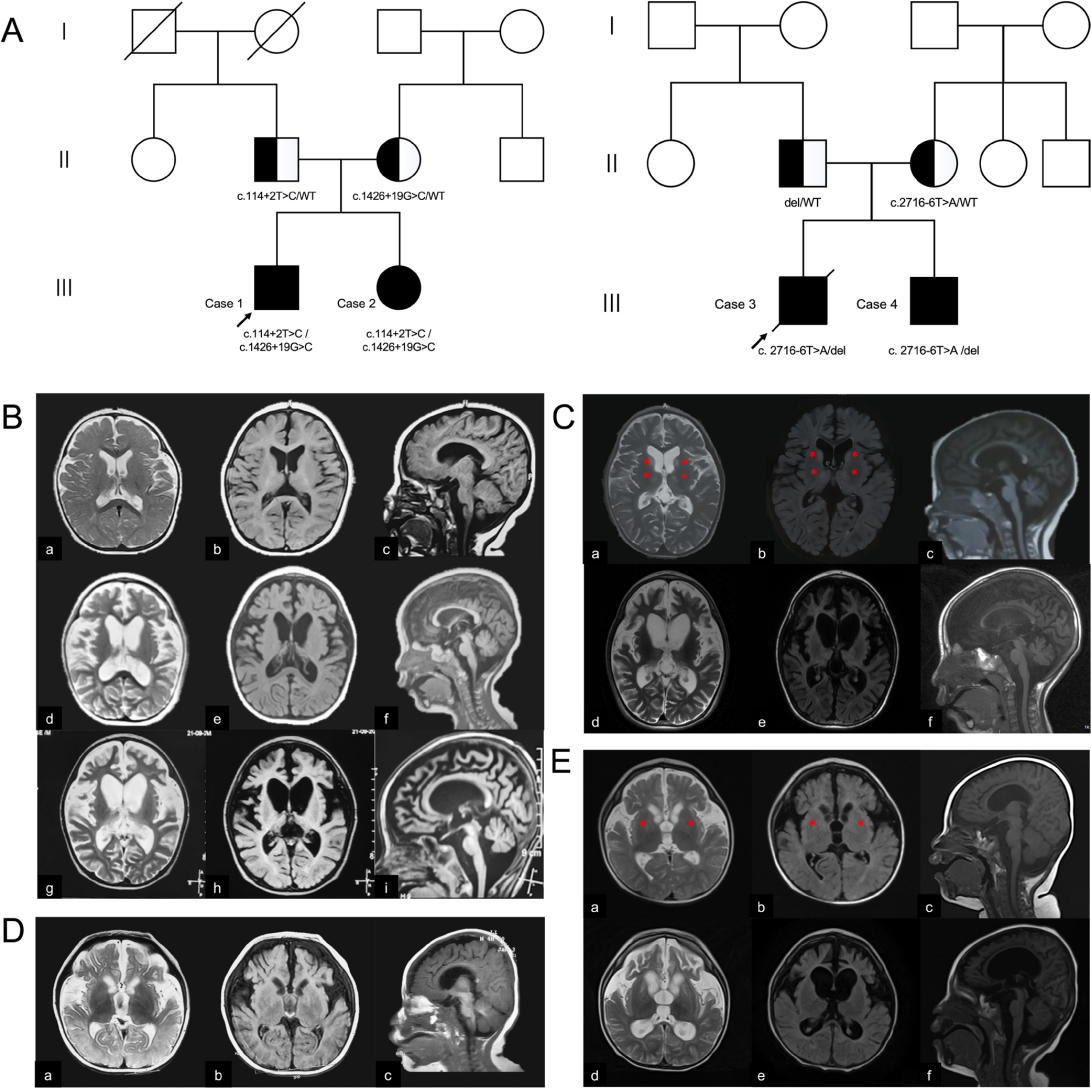
Patient pedigree and neuroimaging. (A) Pedigrees of family 1 (left, case 1 and case 2) and family 2 (right, case 3 and case 4). (B–E) Brain MRI images from four patients showing progressive whole‐brain atrophy with or without hyperintense signal in the basal ganglia region. (B) Brain MRI of case 1 at 10 months (a–c), 15 months (d–f), and 21 months (g–i) showing progressive global cerebral atrophy. (C) Brain MRI of case 2 at 15 months (a–c) and 36 months (d–f) demonstrating progressive global cerebral atrophy with abnormal signal foci in the head of the caudate nucleus and thalamus (a, b; red asterisks). (D) Brain MRI of case 4 at 4 months (a‐c) showing enlarged extracerebral space and a thin corpus callosum. (E) Brain MRI of case 3 at 9 months (a–c) and 12 months (d–f), also showing progressive global cerebral atrophy with abnormal signal foci in the lentiform nucleus (a, b; red asterisks). (a, d, g): Transverse T2‐weighted images; (b, e, h): Transverse FLAIR images; (c, f, i): Sagittal T1‐weighted images.

Case 1 was the first male child of a healthy, nonconsanguineous Chinese Han family, currently aged 8 years 3 months (Figure 1A and Table 1). He was born following an uncomplicated pregnancy and neonatal period. At 6 months of age, he was noted to have developmental milestone delays, including the inability to lift his head. At 10 months of age, a brain MRI revealed bilateral ventricular enlargement and thinning of the corpus callosum (Figure 1B,a–c), but the EEG was normal. He subsequently began rehabilitation therapy. At the age of 1 year and 2 months, he developed a daily series of head nodding. An EEG at this time captured epileptic spasms and interictal atypical hypsarrhythmia. His seizures were not controlled with vigabatrin, valproic acid, topiramate, or levetiracetam. He presented with developmental regression, spasticity, and involuntary movements without epileptic discharge on EEG, though symptoms improved with the administration of benzodiazepines. A second MRI revealed brain and cerebellar atrophy (Figure 1B,d–f). By the age of 1 year and 9 months, his MRI showed progression of brain atrophy (Figure 1B,g–i). A mitochondrial disorder was suspected, but genetic testing, including trio whole‐exome sequencing (WES), CNV sequencing (CNV‐seq), and mitochondrial genome sequencing (mito‐seq), was negative. A mitochondrial cocktail was administered. By age 2 years, his EEG showed that he still experienced epileptic spasms and tonic seizures. He was diagnosed with developmental and epileptic encephalopathy (DEE) and treated with a combination of topiramate and benzodiazepines. The latest EEG (at the age of 5 years) did not capture seizures but showed ongoing multifocal interictal spikes. Currently, he has a severe developmental delay and is wheelchair‐bound. His physical growth is poor (weight: 13 kg, ≤ −3 SD; head circumference: 48 cm, ≤ −3 SD).

**TABLE 1:** Clinical features and genetic information of the four *AFG3L2* patients.

	Case 1	Case 2	Case 3	Case 4
Family	Family 1	Family 1	Family 2	Family 2
Sex	Male	Female	Male	Male
Developmental delay onset	6 months	7 months	6 months	4 months
Epilepsy onset	14 months	14 months	9 months	4 months
Main seizure type	Epileptic spasms, tonic seizures	Epileptic spasms, myoclonic seizures	Epileptic spasms, tonic seizures, tonic–clonic seizures	Epileptic spasms, tonic seizures
EEG	Hypsarrhythmia	Hypsarrhythmia	Hypsarrhythmia	Hypsarrhythmia
Response to ASMs	Refractory	Refractory	Refractory	Refractory
Developmental phenotype	Severe global developmental delay	Severe global developmental delay	Severe global developmental delay	Severe global developmental delay
Last physical examination	Microcephaly, generalized spasticity, dystonia	Microcephaly, generalized spasticity, dystonia	Microcephaly, generalized spasticity	Microcephaly, generalized spasticity, hypotonia
Lactic acid^a^	NA	Elevated, 5.4 mmol/L	Elevated, 2.4 ~ 3.4 mmol/L	Elevated, 2.58 mmol/L
Brain MRI	Progressive whole‐brain atrophy	Progressive whole‐brain atrophy with basal ganglia involvement	Progressive whole‐brain atrophy with basal ganglia involvement	Progressive whole‐brain atrophy
Variants information^b^	c.114 + 2 T > C; c.1426 + 19G > C	c.114 + 2 T > C; c.1426 + 19G > C	g.12359480‐12361132del; c.2176‐6 T > A	g.12359480‐12361132del; c.2176‐6 T > A
Inheritance	Autosomal recessive	Autosomal recessive	Autosomal recessive	Autosomal recessive
Current status	Alive, 8 years	Alive, 3 years	Deceased, 4 years	Alive, 1.5 years

*Note:* The reference range for serum lactate acid is 0.6–2.2 mmol/L.

^a^ The reference range for serum lactate acid is 0.6–2.2 mmol/L.

^b^ The variants are described according to the reference sequence. (NC_000018.10 for genomic reference sequence and NM_006796.3 for coding DNA reference sequence).

Case 2 is the younger sister of case 1, a female aged 3 years 2 months (Figure 1A and Table 1). Her birth history was normal, but she was noted to have a motor developmental delay at 7 months of age due to an inability to sit independently and subsequently received rehabilitation therapy. At the age of 1 year and 2 months, she developed a series of head nodding. An EEG showed interictal atypical hypsarrhythmia and recorded epileptic spasms. Brain MRI demonstrated whole‐brain atrophy with abnormal signal foci in the head of the caudate nucleus and the thalamus (Figure 1C,a–c). She received vigabatrin without benefit, and her psychomotor regression became more pronounced. A diagnosis of DEE was subsequently established. At the age of 1 year and 3 months, an EEG captured a series of epileptic spasms. An ACTH regimen (2 U/kg/day for 21 days) was administered but proved ineffective. Despite being treated with a benzodiazepine and topiramate, her seizures remained uncontrolled. At the age of 3 years and 1 month, an EEG continued to show interictal atypical hypsarrhythmia and captured myoclonic seizures and epileptic spasms. Her brain MRI showed progressive whole‐brain atrophy (Figure 1C,d–f). Genetic tests, including both trio WES and mito‐seq, were negative. At present, she exhibits severe delays in motor, verbal, and social development. Moreover, like her sibling, she has markedly delayed physical growth (weight: 9 kg, ≤ −3 SD; head circumference: 45 cm, ≤ −2 SD). She also presents with spasticity and numerous involuntary movements.

Case 3 is a Chinese Han male, the first child of another nonconsanguineous couple, likewise born after an uncomplicated pregnancy and neonatal course and with no relevant family history (Figure 1A and Table 1). However, severe developmental milestone delays were noted by the age of 6 months. An EEG at this time was normal, but an MRI showed whole‐brain atrophy with abnormal signal foci in the lentiform nucleus (Figure 1E,a–c). He received rehabilitation therapy. At the age of 9 months, he developed seizures characterized by gaze deviation, with or without limb rigidity, occurring 5 to 6 times daily. Shortly thereafter, he began experiencing a series of head nodding, accompanied by worsening psychomotor development. He presented with poor physical growth and microcephaly (weight: 9 kg, ≤ −3 SD; head circumference: 41 cm, ≤ −3 SD), and spasticity. An EEG at this time showed interictal hypsarrhythmia and captured a series of epileptic spasms and focal tonic seizures. An MRI showed brain atrophy, corpus callosum dysplasia, and supratentorial hydrocephalus. He was diagnosed with DEE and received an ACTH regimen (2 U/kg/day for 14 days), which temporarily halted the spasms, but seizures soon recurred. Subsequent treatment with topiramate, benzodiazepines, valproate, and a second ACTH regimen (2 U/kg/day for 21 days) also failed. At 1 year and 4 months, he remained unable to lift his head or sit independently. An EEG at this time captured multiple seizure types, including epileptic spasms, tonic–clonic seizures, asymmetric tonic seizures, and focal seizures, and an MRI showed the progression of brain atrophy and hydrocephalus (Figure 1E,d–f). Genetic testing via trio WES and CNV‐seq did not identify any causative variants. During the last clinical follow‐up, it was reported that he had passed away at the age of 4 years, possibly due to pneumonia.

Case 4 is a male aged 1 year, 7 months, the younger brother of case 3, born after an unremarkable perinatal history (Figure 1A and Table 1). At the age of 4 months, developmental delay was noted. A brain MRI revealed widened extracerebral spaces and a thin corpus callosum (Figure 1D). His EEG showed an interictal hypsarrhythmia and captured epileptic spasms. He was treated with topiramate and benzodiazepines, but his seizures remained uncontrolled. He experienced daily clusters of head nodding. He underwent trio WES, which did not identify any causative variant. At the age of 1 year and 7 months, he was referred to our hospital. At that time, he exhibited severe psychomotor delays. Physical examination revealed poor somatic growth, including microcephaly (weight: 6.5 kg, ≤ −3 SD; head circumference: 39.5 cm, ≤ −3 SD), and spasticity. An EEG captured tonic seizures and epileptic spasms, with interictal multifocal as well as generalized spike‐and‐wave discharges.

### 3.2 Biallelic Variants of *AFG3L2* Identified Through Whole‐Genome Sequencing

Compound heterozygous variants in *AFG3L2* were detected in case 1 and case 2 via whole‐genome sequencing (Figure 1A; Figure S1A,B). The paternal variant (NM_006796.3: c.114 + 2 T > C) is located at the canonical splice donor site of intron 1, with a frequency of < 0.1% in the gnomAD population database. To predict the variant's pathogenicity, we evaluated it using published computational tools. The paternal variant had a Combined Annotation Dependent Depletion (CADD) [11] score of 32, a SpliceAI [12] donor loss score of 0.98, and a Pangolin [13] splice loss score of 0.69, all of which support its pathogenicity. This variant is not listed in the ClinVar database and is classified as pathogenic based on the following criteria: PVS1 + PM2_supporting + PP4. The maternal variant (NM_006796.3: c.1426 + 19G > C) is within intron 11 of *AFG3L2* and absent from the gnomAD database. The variant CADD score was 8.372, the SpliceAI acceptor loss score was 0.17, and the Pangolin splice loss score was 0.07. This variant is not recorded in ClinVar and was classified as a variant of uncertain significance (VUS): PM2_Supporting + PM3 + PP4.

In cases 3 and 4, two *AFG3L2* variants were identified, a 1632 bp deletion and an intronic single‐nucleotide variant (SNV) (Figure 1A and Figure S2A,B). The paternal deletion (NC_000018.10: g.12359480_12361132del) spans exon 7 of *AFG3L2*. This region is associated with a pathogenic deletion variant (#501370) in the DECIPER database. Additionally, two pathogenic loss‐of‐function variants in this region (#1027438; #2192635) have been reported in the ClinVar database. We classified this variant as likely pathogenic based on the 0.9‐point criteria (2C‐1). The maternal variant (NM_006796.3: c.2176‐6 T > A) is located in the last intron of *AFG3L2*, with a frequency of < 0.1% in the gnomAD population database. The variant was predicted to be pathogenic, with a CADD score of 23.0, a SpliceAI acceptor‐gain score of 0.64, and a Pangolin splice‐gain score of 0.43. Moreover, Jin et al. reported a Chinese child with microcephaly, refractory seizures, and cerebral atrophy carrying this variant in compound heterozygosity with p.E612* [10]. We classified this variant as likely pathogenic based on the following criteria: PM2_Supporting + PM3_Strong + PP3 + PP4.

**FIGURE 2:**
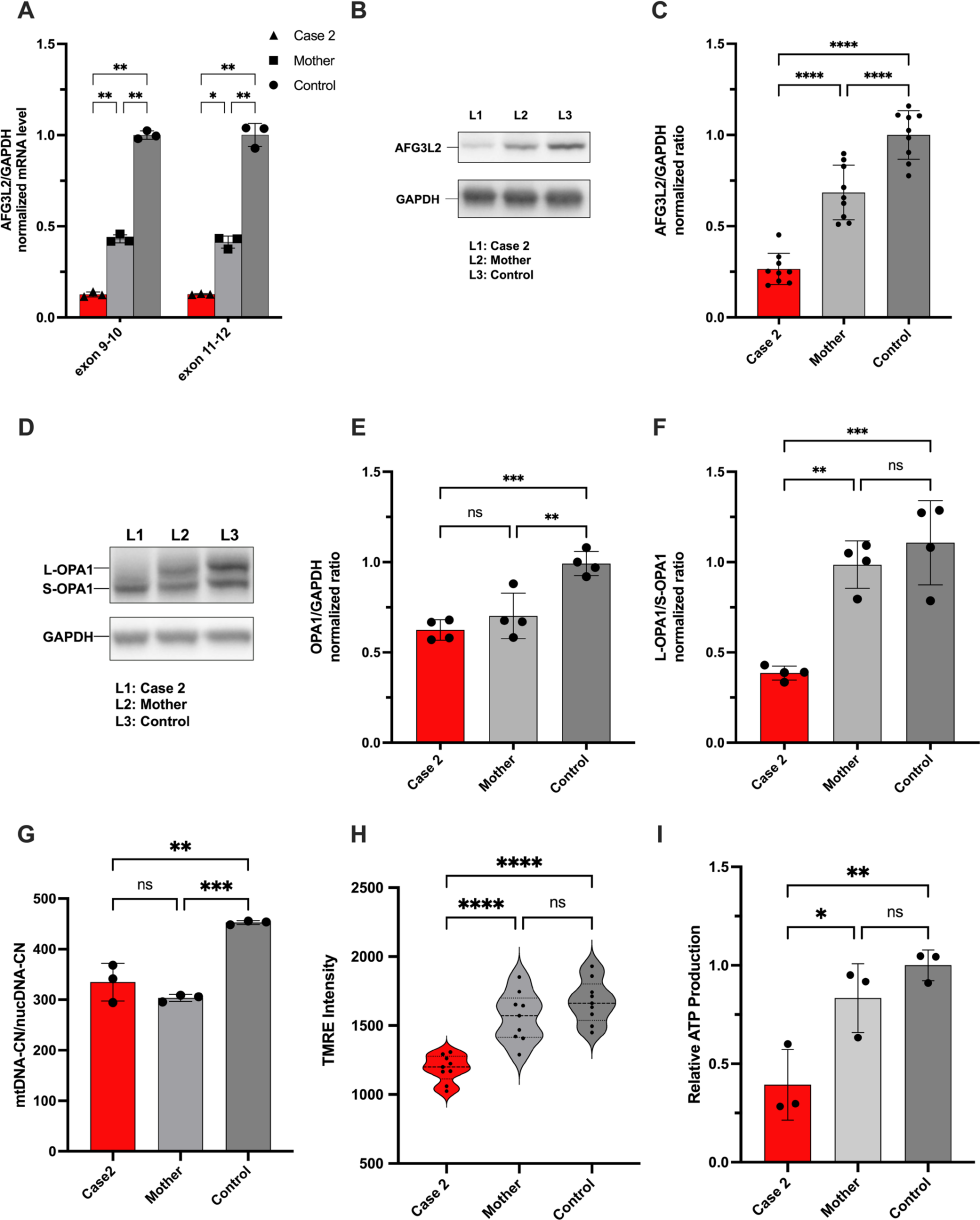
Reduced *AFG3L2* mRNA expression and mitochondrial dysfunction in fibroblasts from case 2 and her mother. (A) Quantitative PCR reveals reduced *AFG3L2* mRNA expression in skin fibroblasts from case 2 and her mother compared to a healthy control; *GAPDH* was used as an internal reference (*n* = 3 per group). (B, C) Western blotting shows decreased AFG3L2 protein expression in skin fibroblasts from case 2 and her mother relative to a healthy control, with GAPDH as the loading control (*n* = 9 per group). (D–F) Western blot analysis shows that total OPA1 protein levels in fibroblasts from case 2 and her mother were significantly reduced compared with a healthy control (E), with no significant difference between the two. GAPDH was used as the loading control. The long‐form‐to‐short‐form OPA1 (L‐OPA1/S‐OPA1) ratio was lower in case 2 than in her mother and the control (F) (*n* = 4 per group). (G) Quantitative PCR shows that the relative mitochondrial DNA (mtDNA) to nuclear DNA (nDNA) copy number was significantly decreased in fibroblasts from both case 2 and her mother compared to the control, with no significant difference between the patient and her parent (*n* = 3 per group). (H) The TMRE‐Mitochondrial Membrane Potential Assay reveals a significant reduction in mitochondrial membrane potential in fibroblasts from case 2 compared to her mother and the control (*n* = 9 per group). (I) ATP bioluminescence assay indicates significantly reduced ATP production in fibroblasts from case 2 compared to her mother and the healthy control (*n* = 3 per group). Data are presented as mean ± SEM. Differences among groups were analyzed by one‐way ANOVA followed by Tukey's multiple comparisons test. ns, no significance; *p < 0.05; **p < 0.01; ***p < 0.001; ****p < 0.0001.

### 3.3 Aberrant Expression and Splicing Events of *AFG3L2* Identified by RNA Sequencing

As part of a prospective clinical trial of 96 families with DEE presenting with an infantile epileptic spasms syndrome phenotype (ChiCTR2100049098), RNA sequencing data were obtained from peripheral blood samples of patients in this study and analyzed to identify expression (by OUTRIDER, *p* < 0.05 and | *Z* | > 3) and splicing (by FRASER, *p* < 0.001, |Z scores| > 2, and |delta‐psi| > 0.15) outlier events. In case 3, an alternative 5′ splicing site event (*p* = 5.4 × 10^−7^, *Z* = −7.24) was detected, which was associated with variant c.2176‐6 T > A. This event results in a 19 bp shortening at the 5′ splice site of *AFG3L2* exon 17 (Figure S2C), consistent with the findings reported by Jin et al. [10]. The same splicing event was observed in the patient's mother. In addition, a skipped exon event (*p* = 5.8 × 10^−5^, *Z* = −4.52), associated with the 1632 bp paternal deletion variant, was identified in case 3 and his father (Figure S2D). OUTRIDER analysis revealed a reduction in *AFG3L2* expression compared to controls (*p* = 0.047, *Z* = −1.89).

For cases 1 and 2, FRASER did not detect aberrant splicing events near variants c.114 + 2 T > C or c.1426 + 19G > C. However, OUTRIDER analysis revealed a significant reduction in *AFG3L2* expression in both cases compared to controls (*p* = 2.09 × 10^−7^, *Z* = −6.51). Furthermore, between exons 1 and 2 of *AFG3L2*, IGV visualization revealed that cases 1 and 2 had 1 and 3 splice junctions, respectively. Their father and mother had 19 and 16 junctions, respectively, while an unrelated control showed 34 junctions (Figure S1C). These findings suggest that aberrant *AFG3L2* transcripts may have been degraded by nonsense‐mediated mRNA decay, thereby limiting the detection of atypical splicing events. We also observed intron retention between exons 11 and 12 of *AFG3L2* in cases 1 and 2 and their mother (Figure S1D). Agarose gel electrophoresis of peripheral blood mRNA from case 1, case 2, and their mother showed specific amplification of *AFG3L2* intron 11, whereas the father's mRNA did not amplify this fragment (Figure S3).

### 3.4 Decreased mRNA and Protein Expression of *AFG3L2* in Skin Fibroblasts

To assess possible effects of variant c.1426 + 19G>C on *AFG3L2*, we isolated and cultured skin fibroblasts from case 2 and her mother. Following stable propagation, qRT‐PCR and WB analysis were performed. A pronounced decrease in *AFG3L2* mRNA expression was observed in fibroblasts from both case 2 (fold change = 0.127, *p* < 0.01) and her mother (fold change = 0.413, *p* < 0.01) (Figure 2A). Furthermore, WB analysis showed a notable reduction in AFG3L2 protein expression in case 2 (fold change = 0.2658, *p* < 0.0001), while a more moderate decrease was observed in her mother (fold change = 0.6849, *p* < 0.0001) (Figure 2B,C).

### 3.5 Altered Mitochondrial Function in Skin Fibroblasts With *AFG3L2* Variants

We conducted functional experiments in skin fibroblasts to measure any changes associated with each *AFG3L2* variant. Given AFG3L2's role in OPA1 proteolytic processing, we first assessed OPA1 expression using WB. We observed a significant reduction in the L‐OPA1/S‐OPA1 ratio in case 2 compared to normal controls (fold change = 0.348, *p* = 0.0003), with no significant change in the mother (Figure 2D,F). Additionally, total OPA1 expression levels were slightly reduced in case 2 and the mother compared to controls (fold change = 0.6244 and 0.7023, *p* = 0.002 and 0.008, respectively, Figure 2E). In addition, we observed aberrant mitochondrial function in the patient's fibroblasts. The mtDNA copy number was lower in fibroblasts from case 2 (mean difference = −117.8, *p* = 0.001) and her mother (mean difference = −149.0, *p* = 0.0004) compared to normal controls (Figure 2G). Furthermore, case 2 fibroblasts demonstrated a marked decrease in MMP (mean difference = −479.9, *p* < 0.0001) and ATP generation (fold change = −0.608, *p* = 0.001) (Figure 2H,I).

**FIGURE 3:**
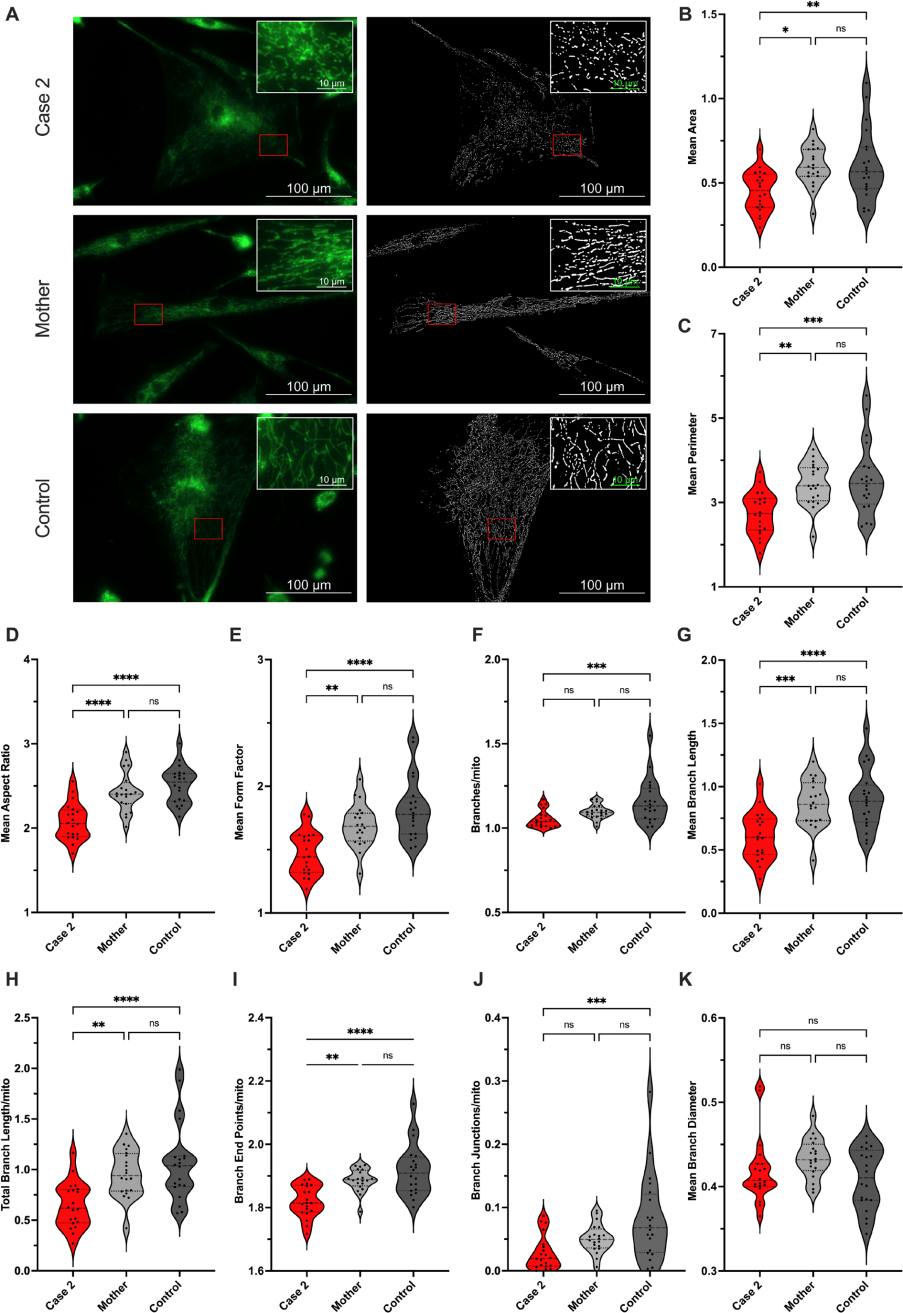
Mitochondrial network morphology in patient‐derived fibroblasts from case 2 and her mother. (A) Fluorescence microscopy images of mitochondria in fibroblasts from case 2, her mother, and a healthy control, labeled with MitoTracker dye (*n* = 20 per group). (B–K) Quantitative analysis of mitochondrial morphology and network in fibroblasts from case 2, her mother, and normal control fibroblasts. Morphological parameters (B–E): Counts are the number of mitochondria per cell; (B) mean area is the total area divided by count. (C) mean perimeter: The total perimeter divided by the count. (D) mean aspect ratio (i.e., length‐to‐width ratio), and (E) mean form factor: The shape measure, where higher values indicate a more elongated mitochondrion. Network parameters (F–K): (F) branches/mito: Number of branches in each mitochondrial skeleton. (G) mean branch length: Total branch length divided by the number of branches. (H) total branch length/mito: Sum of the length of all branches in each mitochondrial skeleton. (I) branch junctions/mito: Number of junctions within each mitochondrial skeleton. (J) branch end points/mito, the total number of endpoints in each mitochondrial skeleton. (K) mean branch diameter. Data are presented as mean ± SEM. Differences among groups were analyzed by one‐way ANOVA followed by Tukey's multiple comparisons test. ns, no significance; * *p* < 0.05; **p < 0.01; ***p < 0.001; ****p < 0.0001.

OPA1 is critical in mammalian mitochondrial fusion, prompting us to investigate the mitochondrial network in patient‐derived fibroblasts. MitoTracker staining showed that fibroblasts from case 2 exhibited reduced mitochondrial mean area (mean difference = −0.149, −0.155, *p* = 0.01, 0.007), perimeter (mean difference = −0.678, −0.806, *p* = 0.003, 0.0005), aspect ratio (mean difference = −0.352, −0.435, *p* < 0.0001, < 0.0001), and form F factor (mean difference = −0.224, −0.354, *p* = 0.002, < 0.0001) compared to the mother and control, respectively (Figure 3A–E). Furthermore, mitochondrial branching complexity was notably diminished in case 2 fibroblasts, with fewer branches (mean difference = −0.042, −0.105, *p* = 0.264, 0.0007), branch junctions (mean difference = −0.022, −0.053, *p* = 0.255, 0.0009), branch endpoints (mean difference = −0.066, −0.097, *p* = 0.002, < 0.0001), and shorter mean branch lengths (mean difference = −0.260, −0.293, *p* = 0.0005, < 0.0001). In contrast, mean branch diameter (mean difference = −0.014, 0.010, *p* = 0.3654, 0.6011) remained consistent (Figure 3F–K). These results indicate substantial mitochondrial network fragmentation in case 2.

**FIGURE 4:**
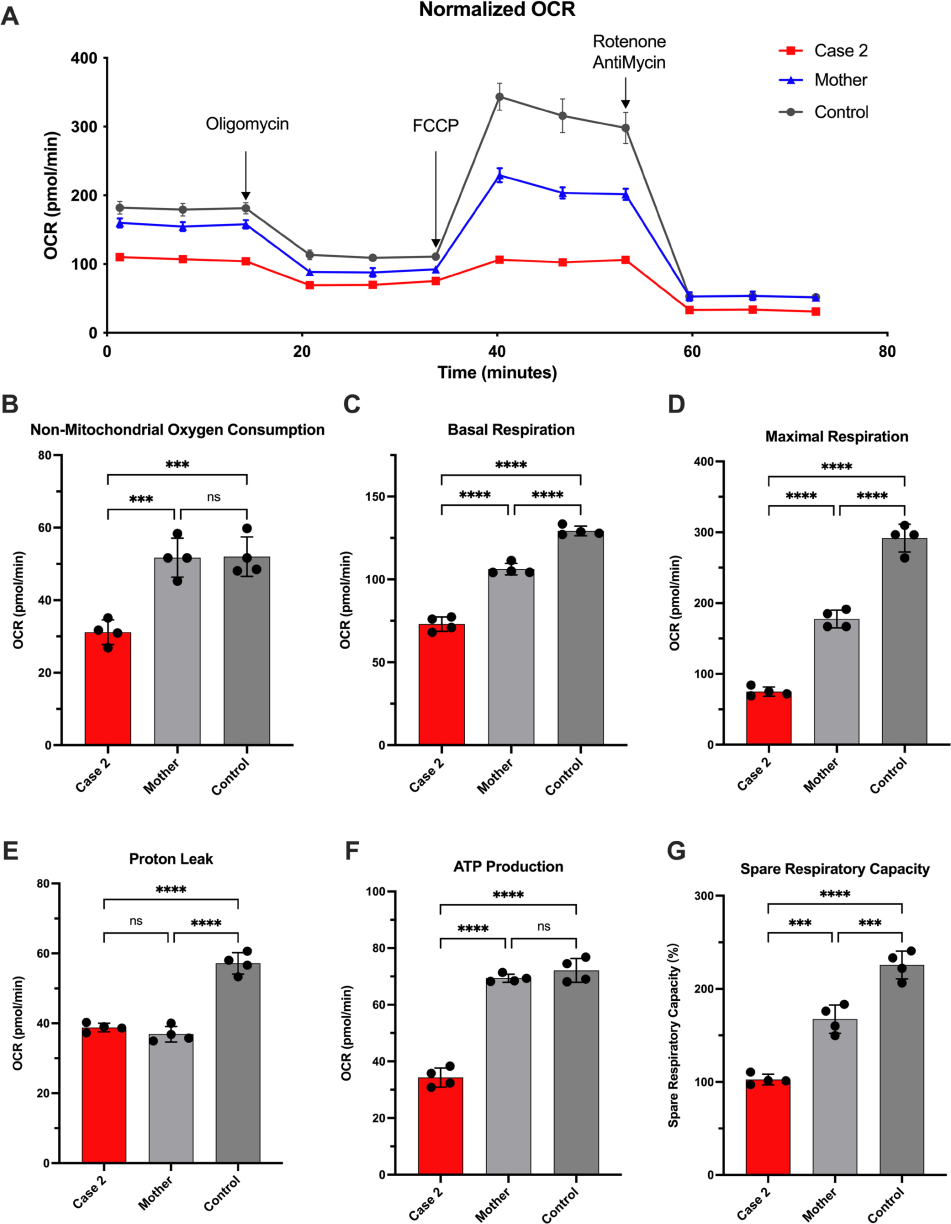
AFG3L2 dysfunction impairs mitochondrial respiration in patient‐derived fibroblasts. (A) Seahorse analysis of the oxygen consumption rate (OCR) in live fibroblasts from case 2, her mother, and a healthy control (*n* = 20 per group). (B–G) Quantification of OCR levels shown as non‐mitochondrial respiration (B), basal respiration (C), maximal respiration (D), proton leak (E), ATP production (F), and spare respiratory capacity (G) are shown. OCR values are normalized to cell count. Data are presented as mean ± SEM. Differences among groups were analyzed by one‐way ANOVA followed by Tukey's multiple comparisons test. ns, no significance; * *p* < 0.05; **p < 0.01; ***p < 0.001; ****p < 0.0001.

Finally, we assessed the oxygen consumption rates (OCR) of each patient's fibroblasts. The Seahorse mito‐stress test revealed significantly reduced OCR levels in cultured fibroblasts from both case 2 and her mother (Figure 4A–G). Specifically, case 2 fibroblasts showed a decline in non‐mitochondrial oxygen consumption (mean difference = −20.85, *p* = 0.0005), basal respiration (mean difference = −56.15, *p* < 0.0001), maximal respiration (mean difference = −216.6, *p* < 0.0001), ATP production (mean difference = −37.79, *p* < 0.0001), and spare respiratory capacity (mean difference = −123.0, *p* < 0.0001). In contrast, maternal fibroblasts exhibited a milder reduction in basal respiration (mean difference = −23.02, *p* < 0.0001), maximal respiration (mean difference = −114.1, *p* < 0.0001), and spare respiratory capacity (mean difference = −58.22, *p* = 0.0003). Case 2 and her mother's fibroblasts also exhibited a reduction in proton leak (mean difference = −18.37, −20.27; *p* < 0.0001, < 0.0001). Together, these results suggest that fibroblasts from both affected individuals had reduced mitochondrial respiratory capacity, with a more severe defect in case 2.

### 3.6 Variant Reclassification

Based on RNA‐seq results and functional assessments of patient‐derived fibroblasts, we reclassified the c.1426 + 19G>C variant from VUS to likely pathogenic, according to the criteria: PS3 + PM2_Supporting + PM3 + PP4. Specifically, PS3 was assigned based on functional evidence from RNA‐seq and fibroblast assays, PM2_Supporting for rarity in population databases, PM3 for detection in trans with another *AFG3L2* variant under a recessive model, and PP4 for the patients' highly specific *AFG3L2*‐related clinical phenotype.

### 3.7 Phenotype and Genotype of *AFG3L2* ‐Related Developmental and Epileptic Encephalopathy

**FIGURE 5:**
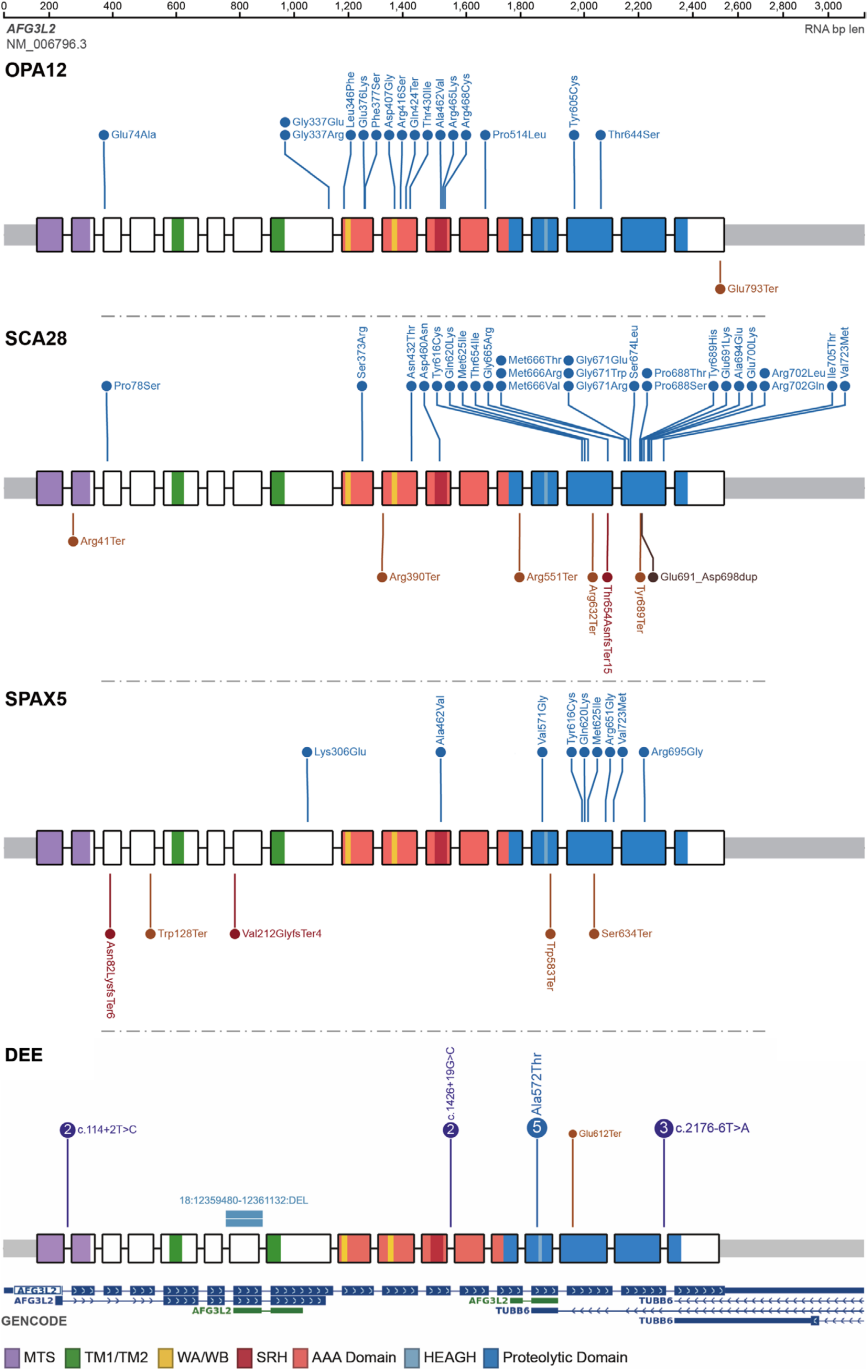
Spatial distribution of disease‐associated variants in the *AFG3L2* gene. Distribution of pathogenic and likely pathogenic variants in *AFG3L2* (NM_006796.3) reported in the ClinVar database and associated with OMIM disease phenotypes, including optic atrophy 12 (OPA12, top panel), spinocerebellar ataxia 28 (SCA28, second panel), spastic ataxia 5 (SPAX5, third panel), and developmental and epileptic encephalopathy (DEE, bottom panel). OPA12‐associated variants were mainly distributed in the AAA region; SCA28‐associated variants showed clustering within the proteolytic domains; SPAX5‐associated biallelic variants involved the proteolytic region as well as truncating variants in other regions. Six distinct *AFG3L2* variants were identified in 10 patients with DEE. All were present in either homozygous or compound heterozygous states, resulting in loss‐of‐function effects. Domain annotations include: MTS, mitochondrial targeting sequence; TM1/TM2, transmembrane domains 1 and 2; WA/WB, Walker A and Walker B motifs; SRH, second region of homology; HEAGH, protease catalytic site. Numbers indicate how many times each variant has been reported.

To date, 10 patients (including the four patients in this study) have been identified worldwide with *AFG3L2*‐related developmental epileptic encephalopathy. A summary of all variants and their corresponding clinical phenotypes is presented in Table 1 and in Table S1; [9, 10]. In all cases, severe developmental delay was evident before seizure onset, with seizures beginning between 6 and 14 months of age. Subtypes of epilepsy included epileptic spasms (*n* = 9), tonic seizures (*n* = 4), and myoclonic seizures (*n* = 2), all of which were drug‐resistant. Interictal EEG commonly showed hypsarrhythmia. Additional key features included severe microcephaly, generalized spasticity, mildly elevated serum lactate, and progressive whole‐brain atrophy. Basal ganglia abnormalities were frequently noted on MRI. The overall prognosis was poor, with four patients deceased and six surviving. In total, six distinct *AFG3L2* variants were identified across these 10 patients (Table S1 and Figure 5), all of which occurred in a homozygous or compound heterozygous state. Functional evidence from previous studies, as well as this study, supports a consistent loss‐of‐function effect for all six variants [1, 10].

### 3.8 Genotype–Phenotype Correlation of *AFG3L2* ‐Related Neurological Disease

More than sixty distinct pathogenic variants in *AFG3L2* have been reported in association with OPA12, SCA28, SPAX5, and DEE (Figure 5 and Table S2). Among the 16 variants associated with OPA12, 15 are missense variants, and one is a null variant; 14 of the 15 missense variants are inherited in an autosomal dominant manner, and 10 are located within the AAA domain. For SCA28, 33 pathogenic variants have been reported, including 25 missense variants and five null variants. Notably, 27 of the 33 variants are located within the proteolytic domain, and 31 are dominantly inherited. Fourteen variants have been associated with SPAX5, all inherited in an autosomal recessive pattern. These include eight missense and six null variants. Among these, six are compound heterozygous missense‐null combinations, and four are either homozygous or compound heterozygous missense‐missense combinations. Six pathogenic variants have been implicated in DEE, including three compound heterozygous null‐null combinations and one homozygous missense variant, all consistent with recessive inheritance.

## 4 Discussion

AFG3L2 is a key subunit of the m‐AAA protease, a mitochondrial complex critical for cellular function [14, 15, 16, 17]. Partial or complete loss of m‐AAA protease activity can compromise mitochondrial integrity and contribute to disease pathogenesis. Previously, *AFG3L2* has been linked to three neurological disorders: OPA12, SCA28, and SPAX5. Biallelic variants in *AFG3L2* have been reported to cause progressive myoclonic epilepsy, typically with seizure onset between 8 and 10 years of age [7, 8]. Recently, Eskandrani et al. described five affected individuals from a consanguineous family who carried homozygous *AFG3L2* mutations and exhibited a severe neurodegenerative phenotype resembling mitochondrial disease. Similarly, Jin et al. reported a patient with two novel compound heterozygous mutations in *AFG3L2*, who presented with severe neurodegeneration and global cerebral atrophy. All six patients described in these studies exhibited early‐onset epilepsy, progressive microcephaly, infantile spasms or myoclonus, and EEG findings including hypsarrhythmia or frequent sharp and spike waves, with or without basal ganglia abnormalities. Functional analyses of these variants were not conducted. In this study, we report four additional Chinese patients from two unrelated families who exhibit clinical features consistent with those of the six previously reported cases [9, 10]. All 10 patients exhibited DEE—a phenotype not yet associated with *AFG3L2*‐related disorders in the OMIM database.

Through a comprehensive review of both previously reported cases and those in our cohort, we observe that heterozygous variants in *AFG3L2* are primarily associated with OPA12 or SCA28, whereas homozygous or compound heterozygous variants are linked to SPAX5 or DEE. Variants in OPA12 are predominantly missense single‐nucleotide variants (SNVs) within the AAA domain, with null variants rarely implicated. In contrast, SCA28‐associated variants are mainly missense changes clustered in the proteolytic domain or null variants located upstream of this region. Biallelic loss‐of‐function variants in *AFG3L2* lead to a severe impairment of protease activity, causing acute mitochondrial dysfunction in developing neurons and resulting in the severe phenotypes characteristic of SPAX5 or DEE. Notably, SPAX5‐related variants in a heterozygous state may present with normal or mild symptoms similar to OPA12 or SCA28 [18, 19, 20, 21, 22]. Most reported SPAX5 cases involve compound heterozygous genotypes—either missense‐null or missense‐missense—with no null‐null combinations documented to date. In contrast, DEE is primarily associated with compound heterozygous null variants, implying a more complete functional loss of *AFG3L2*. Compared to SPAX5, patients with DEE exhibit earlier disease onset, more severe developmental delay, a higher incidence of epileptic spasms, and more extensive brain atrophy affecting regions beyond the cerebellum. These findings suggest different variant combinations exert a distinct impact on mitochondrial function and clinical severity. However, further experimental studies are needed to validate these observations.

The connection between AFG3L2 dysfunction and epilepsy is not fully elucidated, but likely involves impaired protein synthesis, accumulation of misfolded proteins, mitochondrial fragmentation, and disrupted calcium handling, collectively compromising neuronal viability and axonal integrity [5, 6]. Our study and previous studies revealed that impaired AFG3L2 function can disrupt mitochondrial ribosome biogenesis and lead to OMA1 accumulation, which results in excessive cleavage of L‐OPA1 and impaired mitochondrial fusion [23, 24, 25]. These changes lead to mitochondrial fragmentation, reduced respiratory chain activity, diminished ATP production, and elevated ROS. Subsequent loss of mitochondrial membrane potential and opening of the permeability transition pore promote apoptosis or necrosis [26]. Mitochondrial defects may also perturb glutamate metabolism, leading to excitotoxic synaptic accumulation and neuronal injury [27, 28]. Dysregulated calcium buffering could contribute to abnormal neuronal excitability [29]. Although neuronal samples were unavailable due to limited access to clinical specimens, functional validation was performed in patient‐derived fibroblasts. This model provides an accessible system for assessing disease‐relevant mitochondrial abnormalities and has been widely used in studies of neurological disorders [30]. Nevertheless, fibroblasts cannot fully recapitulate neuronal vulnerability. Further mechanistic studies using iPSC‐derived neuronal models or brain organoids will be required to clarify how *AFG3L2* dysfunction contributes to epileptic encephalopathy.

Notably, although all four patients initially underwent WES and CNV‐seq without detecting pathogenic variants, subsequent WGS identified four candidate variants, including noncoding splice‐region variants and an intragenic deletion. This finding emphasizes the diagnostic advantage of WGS in unresolved DEE cases. Compared with WES, WGS can detect noncoding and deep intronic variants with potential splice‐altering effects, which are generally beyond the scope of exon‐focused sequencing. In addition, WGS outperforms standard CNV‐seq and WES‐based CNV detection in resolving small intragenic CNVs, particularly deletions smaller than 10 kb [31, 32, 33]. In this study, WGS identified a small *AFG3L2* intragenic deletion at approximately kilobase‐level resolution, supporting its clinical utility in patients with high clinical suspicion and uninformative prior testing. Subsequent RNA‐seq and functional analyses of patient‐derived fibroblasts further supported the pathogenic relevance of these WGS‐identified variants.

This study has several limitations. First, because we only evaluated OPA1 levels and no other m‐AAA protease substrates, our assessment of protease activity was not comprehensive. Second, although patient‐derived fibroblasts confirmed m‐AAA dysfunction and mitochondrial respiratory defects, the lack of neuronal models, such as patient‐derived iPSC‐derived neurons, limits confirmation of these mitochondrial phenotypes in a disease‐relevant neural context. Fibroblasts may not fully recapitulate neuron‐specific vulnerability, including synaptic mitochondrial trafficking, activity‐dependent energy demand, and neuronal calcium homeostasis. Furthermore, using patient cells means the contribution of the specific variant cannot be isolated from the patient's genetic background. Finally, the small cohort size warrants larger studies to more accurately delineate the clinical spectrum of *AFG3L2*‐related developmental and epileptic encephalopathy. Future studies leveraging large‐scale resources and international case‐matching platforms, such as GeneMatcher, may help identify additional patients with biallelic coding‐region *AFG3L2* variants and further refine genotype–phenotype correlations.

## 5 Conclusion

In this study, we identified an autosomal recessive *AFG3L2*‐related phenotype distinct from those currently described in the OMIM database. The affected individuals exhibited DEE, specifically infantile epileptic spasms syndrome, featuring severe developmental delay, microcephaly, infantile‐onset intractable epilepsy, developmental regression, and progressive cerebral atrophy with or without basal ganglia involvement on MRI. Combined transcriptomic and functional analyses indicated that a complete loss of *AFG3L2* function constitutes the core pathogenic mechanism underlying this disorder. These findings broaden the clinical and genetic spectrum of *AFG3L2*‐related disorders and underscore the utility of integrated genomic and transcriptomic sequencing in diagnosing DEE of unknown etiology.

## Author Contributions

Zou Pan and Li Yang wrote the manuscript; Zou Pan, Haiyan Tang, Chen Chen, and Qian Yang contributed to the acquisition and analysis of data; Zou Pan and Qian Yang drafted the text and prepared the figures; Zhanwei Zhang, Tenghui Wu, Fang He, and Fangyun Liu contributed to the acquisition of data; Jing Peng contributed to the conception and design of the study.

## Funding

This study was supported by the Natural Science Foundation of Hunan Province (Grant No. 2023JJ30963) and the China Association Against Epilepsy (CAAE) (Grant No. CJ‐2022‐015).

## Ethics Statement

This study was approved by the Ethics Committee of Xiangya Hospital, Central South University (#201603205).

## Consent

Informed consent was obtained from the guardians of all the children.

## Conflicts of Interest

The authors declare no conflicts of interest.

## Supporting information


**Data S1:** Supporting information and methods.

**Figure S1:** Genomic and transcriptomic analysis of *AFG3L2* variants in cases 1 and 2.
**Figure S2:** Genomic and transcriptomic analysis of AFG3L2 variants in cases 3 and 4.
**Figure S3:** Aberrant retention of intron 11 in AFG3L2 transcripts.

**Table S1:** Phenotype and genotype information for *AFG3L2* patients with early‐onset development epileptic encephalopathy.

**Table S2:** Molecular genotype of *AFG3L2*‐related neurological disease.

## Data Availability

The data that support the findings of this study are available from the corresponding author upon reasonable request.
