# Supplementary material for: Loss of Function of AFG3L2 Leading to Developmental and Epileptic Encephalopathy
Source: CNS Neurosci Ther. 2026 Jul 7;32(7):e71013. doi: 10.1002/cns.71013 (PMC13340136; doi:10.1002/cns.71013)
Supplement: Supplementary file 1 — Data S1: Supporting information and methods. [file CNS-32-e71013-s003.docx]

**Supplement S1. Supplementary Materials and Methods**

**Clinical Data and Ethical Compliance**

Patients' clinical information was collected, including demographic data, clinical features, auxiliary examination, and neuroimaging. The diagnostic criteria for IESS were based on the requirements of the International League Against Epilepsy (ILAE) in 2022(1).

**Whole Genome Sequencing (WGS) and RNA Sequencing**

Genomic DNA was extracted from peripheral blood using the QIAamp DNA Blood Mini Kit (51104, QIAGEN). DNA integrity and quality was assessed using the Qubit 4 Fluorometer (Thermo Fisher). Libraries were prepared using the DNA PCR-Free Library Prep Kit (Illumina), then sequenced on an Illumina NovaSeq 6000 platform.

RNA was extracted from peripheral blood mononuclear cells using the QIAamp RNA Blood Mini Kit (52304, QIAGEN). RNA integrity and quality was assessed using the Qubit 4 Fluorometer (Thermo Fisher). Library preparation was performed with the Illumina Stranded Total RNA Prep with Ribo-Zero Plus Kit (Illumina), then sequenced on an Illumina NovaSeq 6000 platform.

**Data Analysis for WGS and RNA Sequencing**

Genomic sequencing reads were aligned to the human reference genome (GRCh38) using the Burrows-Wheeler Aligner (BWA, v0.7.17). Germline variant calling for single-nucleotide variants (SNVs) and insertions and deletions (Indels) was performed using the Sentieon (version 202,010.02) pipeline. Variants were called by GATK HaplotypeCaller and annotated using ANNOVAR and SnpEff (2, 3). Copy number variants (CNVs) were detected using the CNVkit pipeline (4). Structural variants (SVs) were identified using both the Delly and Manta pipelines, and annotated using AnnotSV (5-7).

RNA sequencing reads were aligned to the GRCh38 reference genome using STAR v2.7.6a (8) with the two-pass alignment option, which enables sensitive junction discovery. Aberrant gene expression was identified using OUTRIDER (v1.12.0) (9), with candidate outliers determined based on *p* < 0.05 and absolute Z scores, | Z | > 3. Abnormal splicing events were analyzed using FRASER (v1.7.0)(10), with a significance threshold of *p* < 0.001, |Z scores| > 2, and |delta-psi| > 0.15.

DNA-RNA sample concordance was assessed using PLINK (11), with the identity by descent metric threshold of PI_HAT > 0.8. RNA outlier events were evaluated alongside genomic variant data to identify putative causal variants. More specifically, we identified compound heterozygote genotypes associated with aberrant transcriptomic phenotypes. Variant interpretation was conducted according to American College of Medical Genetics and Genomics (ACMG) guidelines (12). WGS and RNA sequencing data were visualized using the Integrative Genomics Viewer.

**Fibroblast Cell Line Isolation and Culture**

Skin fibroblast cells from the upper arm were biopsied under a local anesthesia procedure from the patient, the patient's parents, and a healthy control (Chinese Han, female, age of 30 years). The skin tissues were treated with the whole skin dissociation kit (Miltenyi, #130-101-540). Subsequently, the obtained cells were cultured in high-glucose (5.5 g/L) Dulbecco's Modified Eagle Medium (DMEM, Gibco) containing 10% fetal bovine serum, 1.5% GlutaMax supplement (Gibco), 1% Penicillin-Streptomycin (Gibco), and 5 ng/ml human fibroblast growth factor protein (MCE).

**Quantitative Real-Time Polymerase Chain Reaction and Western Blotting**

Total RNA was extracted as previously described, and cDNA was synthesized using a HiScript cDNA synthesis kit (Vazyme; R223-01). Quantitative real-time polymerase chain reaction (qRT-PCR) was performed using the SYBR Green Master Mix (Transgene). The primer pairs used are as follows:

| Target | Forward Primer | Reverse Primer |
| --- | --- | --- |
| human *AFG3L2* exon 9-10 | 5'-GCCATTCTCACTGGTCCTCC-3' | 5'-ATTCTTCCGAGCAAGGGCAA-3' |
| human *AFG3L2* exon10-12 | 5'-CTGCTGGTGGAGATGGATGG-3' | 5'-GTCCAGTTTTAGCGGTCGGA-3' |

Western blotting (WB) was conducted as previously described (13). Antibodies used in experiments are as follows:

Primary antibodies:

| Antibody | Catalog No. | Supplier | Dilution |
| --- | --- | --- | --- |
| AFG3L2 | 14631-1-AP | Proteintech | 1:5,000 |
| OPA1 | 27733-1-AP | Proteintech | 1:2,000 |
| GAPDH | 60004-1-Ig | Proteintech | 1:50,000 |

Secondary antibodies:

| Antibody | Catalog No. | Supplier | Dilution |
| --- | --- | --- | --- |
| Goat anti-Rabbit IgG (H + L) Poly-HRP Secondary Antibody | 32260 | Invitrogen | 1:10,000 |
| Goat anti-Mouse IgG (H + L) Poly-HRP Secondary Antibody | 32230 | Invitrogen | 1:10,000 |

**Mitochondrial Morphology Analysis**

Mitochondrial network structure was visualized under fluorescence microscopy using MitoTracker Green (Invitrogen, M7514). Briefly, 10,000 fibroblast cells were seeded in a glass-bottom culture dish (Biosharp) and cultured for 24 hours. Before imaging, cells were washed with pre-warmed DMEM and incubated with 100 nM MitoTracker for 45 minutes. After rinsing twice with PBS, over 20 different stained fibroblast cells in three culture replicates were photographed using a fluorescent microscope (Axio Scope A1, Zeiss, Germany). Mitochondrial morphology was assessed by calculating morphometric parameters and network connectivity using the "mitochondrial analyzer" plugin in Fiji software (14).

**Mitochondrial DNA Copy Number Relative Quantification**

Nuclear DNA (nDNA) and mitochondrial DNA (mtDNA) were extracted using the DNA Extraction Kit (Transgene, EE101-01). The relative copy number of mitochondrial DNA was quantified using the Human Mitochondrial DNA Monitoring Primer Set (Takara, #7246).

**ATP Bioluminescence Assessment**

ATP concentration was measured using the ATP Bioluminescence Assay Kit CLS II (Roche; 11699695001). In brief, 20,000 fibroblast cells were harvested and lysed by incubation in 9 volumes of boiling 100 mM Tris, 4 mM EDTA (pH 7.75). An equal volume of luciferase reagent was then added to the lysate. Luminescence was immediately measured using a Synergy H1 microplate reader (BioTek) with an integration time of 10 seconds. ATP concentrations were determined by referencing a standard curve.

**Mitochondrial Membrane Potential (MMP) Assessment**

MMP levels were assessed using the TMRE Mitochondrial Membrane Potential Assay Kit (Abcam; ab113852). Briefly, 10,000 fibroblast cells were seeded into a black, clear-bottom 96-well microplate (Beyotime; FCP965) and cultured for 3 hours under standard cell culture conditions. Subsequently, 200 nM TMRE working solution was added to each well and incubated for 30 minutes under standard cell culture conditions. After rinsing three times with PBS, 100 μl of PBS containing 0.2% BSA was added to each well, and the fluorescence intensity was measured using a Synergy H1 microplate reader with excitation/emission wavelengths of 549/575 nm.

**Oxygen Consumption Measurement**

Fibroblast cells' oxygen consumption rates (OCR) were measured using the Seahorse Bioscience XF-96 extracellular flux analyzer (Agilent). Briefly, 20,000 fibroblast cells were seeded into a 96-well plate. OCR was detected under basal conditions, followed by treatment with oligomycin, carbonyl cyanide 4-(trifluoromethoxy) phenylhydrazone (FCCP), rotenone, and antimycin A. A detailed protocol is provided in the following table. A CCK-8 assay (MCE) was performed to normalize the OCR rates to cell counts. OCR values are expressed in pmol/min.

| Step | Drug | Concentration |
| --- | --- | --- |
| Injection 1 | Oligomycin | 1.5 µM |
| Injection 2 | FCCP | 2 µM |
| Injection 3 | Rotenone  Antimycin A | 1 µM  1 µM |

**Systematic literature review**

We conducted a systematic literature review focused on *AFG3L2*-related developmental and epileptic encephalopathy by searching the PubMed database using the keywords "AFG3L2", "epilepsy", and "epileptic encephalopathy". All publications were screened for case series or cohort studies reporting clinical data on patients with pathogenic or likely pathogenic variants in *AFG3L2*. For identified cases, we systematically extracted detailed information on clinical features, molecular genetics, and functional studies from both previously reported and newly identified individuals with *AFG3L2*-related developmental epileptic encephalopathy. In addition, the phenotype and genotype information for pathogenic variants associated with OPA12, SCA28, and SPAX5 were collected and summarized from the ClinVar database, and these variants underwent further manual review by searching the published literature.

**Statistical Methods**

Statistical analyses were performed using one-way ANOVA to evaluate differences among multiple groups, with calculations conducted in SPSS 23.0. A *p*-value < 0.05 was considered statistically significant. Data are presented as individual data points or as mean ± SEM.

**References**

1. Zuberi SM, Wirrell E, Yozawitz E, Wilmshurst JM, Specchio N, Riney K, et al. ILAE classification and definition of epilepsy syndromes with onset in neonates and infants: Position statement by the ILAE Task Force on Nosology and Definitions. Epilepsia. 2022;63(6):1349-97.

2. Wang K, Li M, Hakonarson H. ANNOVAR: functional annotation of genetic variants from high-throughput sequencing data. Nucleic Acids Res. 2010;38(16):e164.

3. Cingolani P, Platts A, Wang le L, Coon M, Nguyen T, Wang L, et al. A program for annotating and predicting the effects of single nucleotide polymorphisms, SnpEff: SNPs in the genome of Drosophila melanogaster strain w1118; iso-2; iso-3. Fly (Austin). 2012;6(2):80-92.

4. Talevich E, Shain AH, Botton T, Bastian BC. CNVkit: Genome-Wide Copy Number Detection and Visualization from Targeted DNA Sequencing. PLoS Comput Biol. 2016;12(4):e1004873.

5. Rausch T, Zichner T, Schlattl A, Stutz AM, Benes V, Korbel JO. DELLY: structural variant discovery by integrated paired-end and split-read analysis. Bioinformatics. 2012;28(18):i333-i9.

6. Chen X, Schulz-Trieglaff O, Shaw R, Barnes B, Schlesinger F, Kallberg M, et al. Manta: rapid detection of structural variants and indels for germline and cancer sequencing applications. Bioinformatics. 2016;32(8):1220-2.

7. Geoffroy V, Lamouche JB, Guignard T, Nicaise S, Kress A, Scheidecker S, et al. The AnnotSV webserver in 2023: updated visualization and ranking. Nucleic Acids Res. 2023;51(W1):W39-W45.

8. Dobin A, Davis CA, Schlesinger F, Drenkow J, Zaleski C, Jha S, et al. STAR: ultrafast universal RNA-seq aligner. Bioinformatics. 2013;29(1):15-21.

9. Brechtmann F, Mertes C, Matuseviciute A, Yepez VA, Avsec Z, Herzog M, et al. OUTRIDER: A Statistical Method for Detecting Aberrantly Expressed Genes in RNA Sequencing Data. Am J Hum Genet. 2018;103(6):907-17.

10. Mertes C, Scheller IF, Yepez VA, Celik MH, Liang Y, Kremer LS, et al. Detection of aberrant splicing events in RNA-seq data using FRASER. Nat Commun. 2021;12(1):529.

11. Purcell S, Neale B, Todd-Brown K, Thomas L, Ferreira MA, Bender D, et al. PLINK: a tool set for whole-genome association and population-based linkage analyses. Am J Hum Genet. 2007;81(3):559-75.

12. Richards S, Aziz N, Bale S, Bick D, Das S, Gastier-Foster J, et al. Standards and guidelines for the interpretation of sequence variants: a joint consensus recommendation of the American College of Medical Genetics and Genomics and the Association for Molecular Pathology. Genet Med. 2015;17(5):405-24.

13. Duan H, Pan C, Wu T, Peng J, Yang L. MT-TN mutations lead to progressive mitochondrial encephalopathy and promotes mitophagy. Biochim Biophys Acta Mol Basis Dis. 2024;1870(4):167043.

14. Hemel I, Engelen BPH, Luber N, Gerards M. A hitchhiker's guide to mitochondrial quantification. Mitochondrion. 2021;59:216-24.
