# Supplementary material for: Loss of Function of AFG3L2 Leading to Developmental and Epileptic Encephalopathy
Source: CNS Neurosci Ther. 2026 Jul 7;32(7):e71013. doi: 10.1002/cns.71013 (PMC13340136; doi:10.1002/cns.71013)
Supplement: Supplementary file 2 — Figure S1: Genomic and transcriptomic analysis of AFG3L2 variants in cases 1 and 2. Figure S2: Genomic and transcriptomic analysis of AFG3L2 variants in cases 3 and 4. Figure S3: Aberrant retention of intron 11 in AFG3L2 transcripts. [file CNS-32-e71013-s001.docx]

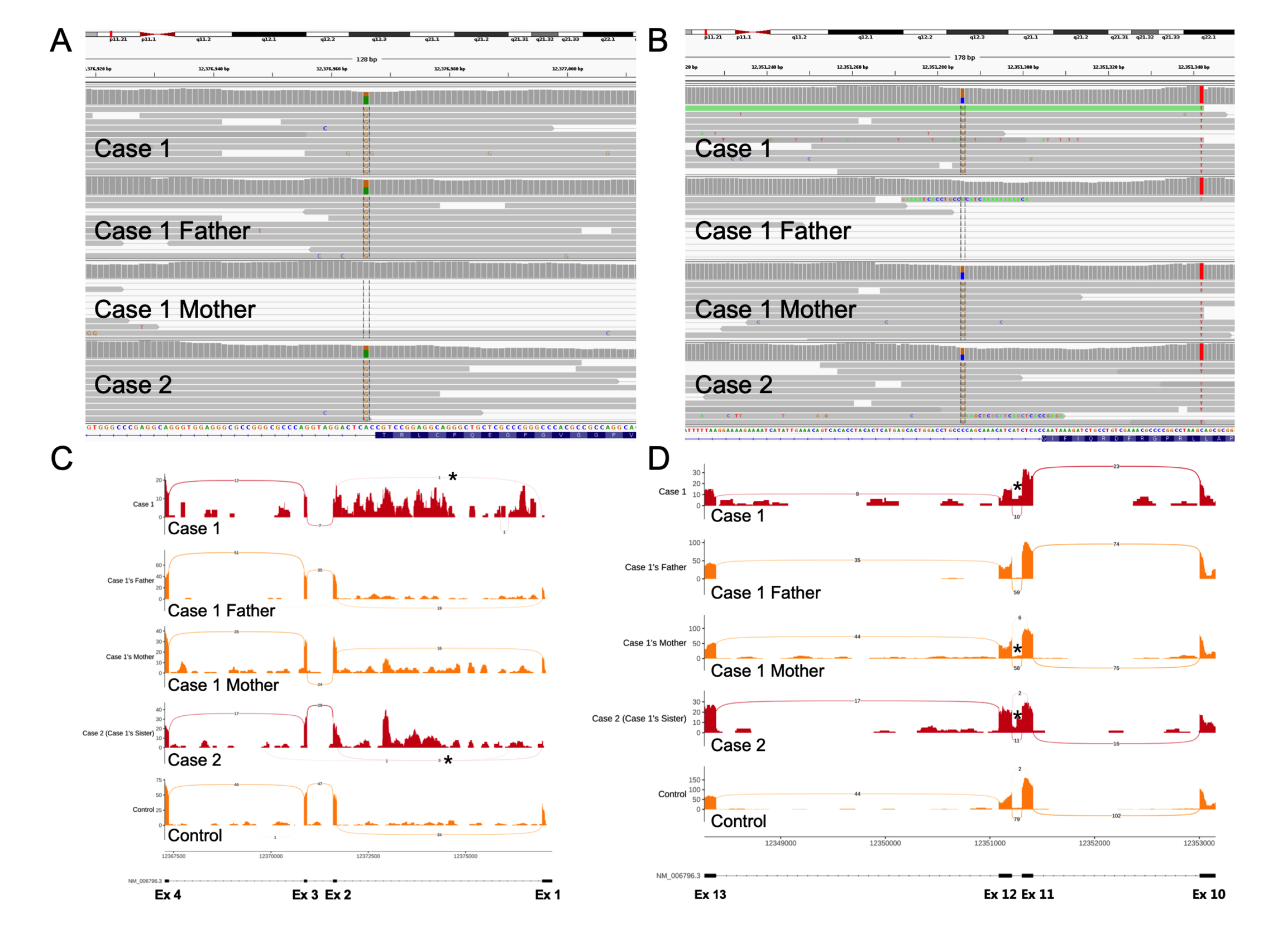


**Figure S1. Genomic and transcriptomic analysis of *AFG3L2* variants in case 1 and case 2.**

(A, B) IGV visualization of read alignments from whole genome sequencing shows compound heterozygous *AFG3L2* variants (NM_006796.3: c.114+2T>C and c.1426+19G>C) in case 1 and case 2, inherited from the heterozygous father and heterozygous mother, respectively. (C) Sashimi plots of peripheral blood RNA-seq in the *AFG3L2* exons 1–4 region show reduced junction read counts between exon 1 and 2 in case 1, case 2, and their father, compared to the mother and a healthy control. (D) Sashimi plots of the *AFG3L2* exons 10–13 region show intron retention between exons 11 and 12 in case 1 and case 2, and their mother, but not in their father or a healthy control.


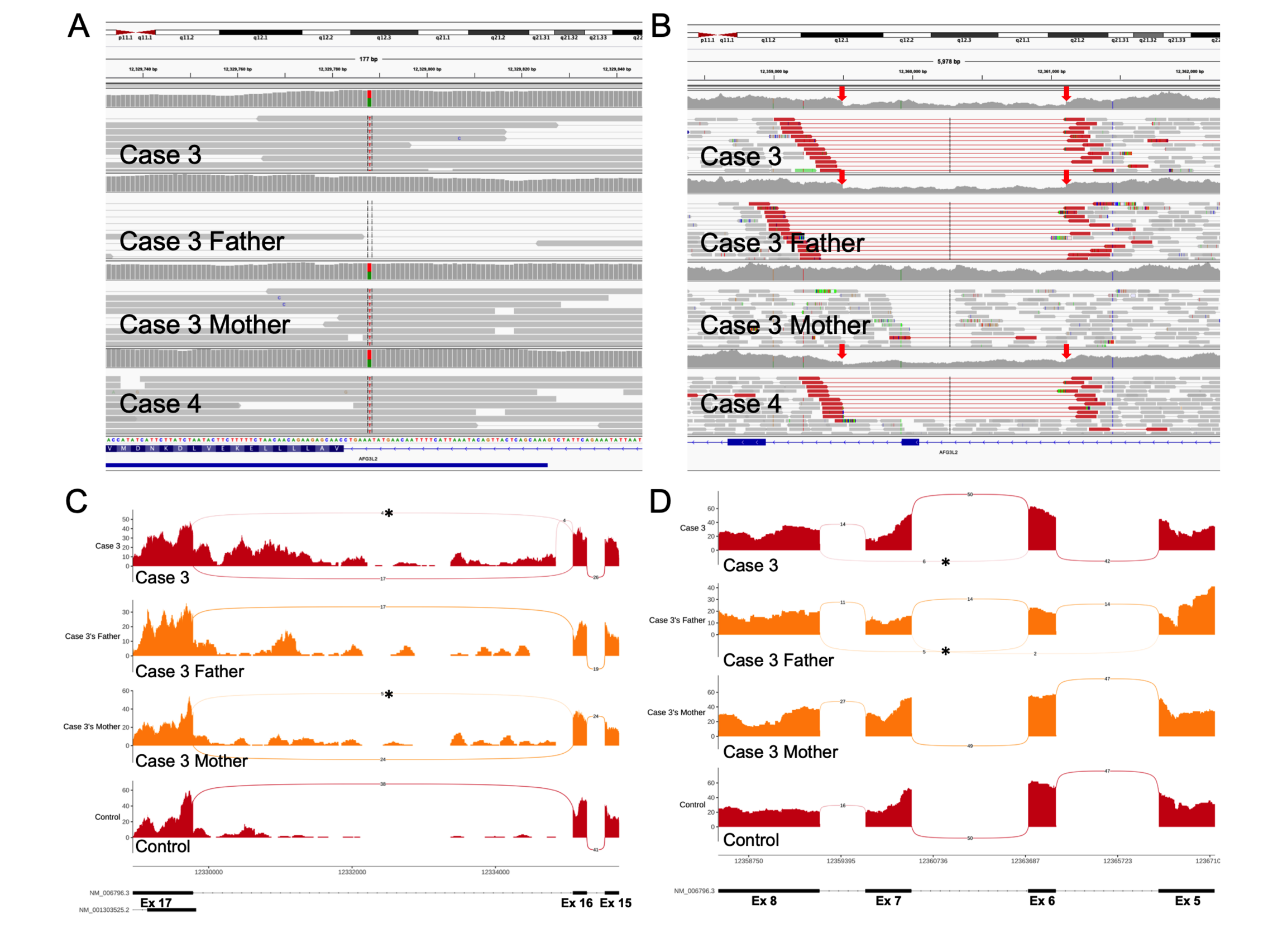


**Figure S2. Genomic and transcriptomic analysis of *AFG3L2* variants in case 3 and case 4**

(A, B) IGV visualization of read alignments from whole genome sequencing (WGS) shows a heterozygous *AFG3L2* variant in case 3 and case 4: NM_006796.3: c.2176-6T>A inherited from the heterozygous mother, and a ~1.6 kb deletion (NC_000018.10: g.12359480_12361132del) inherited from the heterozygous father. (C) Sashimi plots of peripheral blood RNA-seq in the *AFG3L2* exons 15–17 region show a 19 bp shortening at the 5′ splice site of exon 17 in case 3 and his mother compared to his father and a healthy control. (D) Sashimi plots of the *AFG3L2* exons 5–8 region show skipping of exon 7 in case 3 and his father, but not in his mother or the control. RNA sequencing was not performed for case 4, as he was enrolled after the study’s recruitment window had closed.


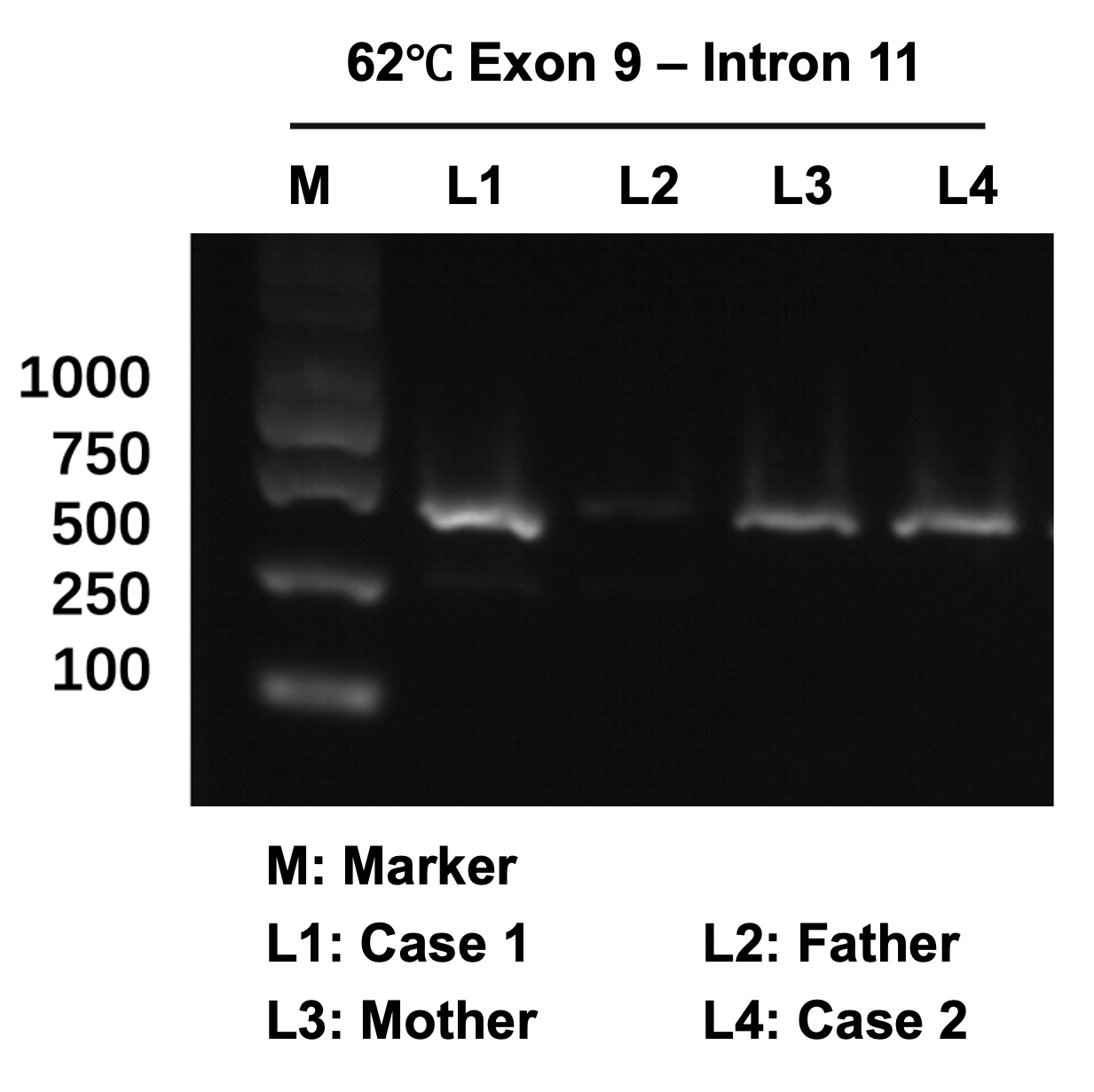


**Figure S3. Aberrant retention of intron 11 in *AFG3L2* transcripts.**

Agarose gel electrophoresis showing intron 11 retention in *AFG3L2* transcripts in case 1 (Lane 1), case 2 (Lane 4), and their mother (Lane 3), but not in their father (Lane 2).
